# Microbial Primer: agr-mediated quorum sensing in Gram-positive pathogens

**DOI:** 10.1099/mic.0.001590

**Published:** 2025-07-31

**Authors:** Paul Williams

**Affiliations:** 1 Biodiscovery Institute and School of Life Sciences, University of Nottingham, University Park, Nottingham, NG7 2RD, UK

**Keywords:** accessory gene regulator (*agr*), autoinducing peptide, Gram-positive, quorum sensing, *Staphylococcus aureus*

## Abstract

Gram-positive bacteria commonly employ autoinducing peptide (AIP) signal molecules to co-ordinate gene expression at the population level. This primer provides a basic overview of *agr*-dependent quorum sensing systems and outlines how AIPs are produced and sensed, what they control and the importance of *agr* for both inter-bacterial and host–pathogen interactions.

## How do bacterial cells communicate?

Although unicellular, bacteria can behave in a multicellular manner driven by individual cells co-operating to swarm, form biofilms, conjugate, sporulate, produce secondary metabolites, cause infections and adapt to specialized environmental niches. Apart from cell-to-cell contact, the production, release and sensing of small diffusible molecules acting as inter-cellular signals offer the most obvious means for individual bacterial cells to co-ordinate their activities. Bacteria synchronize gene expression at a population level through cell-cell communication. The first hints that such inter-cellular signalling existed in bacteria started to emerge in the 1960s and 1970s with respect to genetic competence in *Streptococcus pneumoniae*, streptomycin biosynthesis and sporulation in *Streptomyces griseus* and autoinduction of bioluminescence in marine vibrios.

The realization that cell-cell communication in bacteria was widespread did not become mainstream until the 1990s, when diverse Gram-negative bacteria were discovered to produce *N*-acylhomoserine lactone (AHL) autoinducers synthesized via LuxI proteins and sensed via LuxR response regulators. The term ‘quorum sensing’ (QS) was coined with respect to LuxRI/AHL-type cell-cell communication systems to describe activities that can be performed efficiently only by a sufficiently dense population of bacteria where the minimal unit is a ‘quorum of bacteria’. The quorum is not a fixed number of cells since it depends on the relative rates of synthesis and turnover of the autoinducer and on local environmental conditions. QS is therefore only one environmental parameter (e.g. temperature, pH, osmolarity, oxidative stress and nutrient deprivation) that bacterial cells integrate via gene regulatory networks to determine their optimal survival strategies. Bacterial populations may also exhibit phenotypic heterogeneity in that not all cells in the population will respond to a QS signal such that this aids division of labour and specialization. QS also raises questions with respect to social evolution, fitness and the benefits associated with costly co-operative behaviours. Inhibition of QS has therapeutic potential in the context of infection prevention and control.

Bacteria synthesize a wide range of chemically distinct QS signal molecules. Gram-positive bacteria do not naturally produce AHLs but mostly employ small, linear, modified or cyclic peptides for QS signalling (see https://quorumpeps.ugent.be/). These are proteolytically processed from larger peptides and sensed either at the outer surface of their cytoplasmic membranes or internalized via transporters to gain access to their cytoplasmic receptors. Such peptides are usually referred to as autoinducing peptides (AIPs). Numerous species of streptococci, bacilli and enterococci employ linear AIPs to control phenotypes, including competence induction, virulence factors, bacteriocin production and biofilm formation. This primer will focus on the accessory gene regulator (*agr*) system that employs cyclic AIPs as QS signal molecules and regulates gene expression in Gram-positive pathogens, including staphylococci, clostridia, enterococci and *Listeria* (Table 1).

**Table 1.** Examples of *agr* QS systems and their cognate cyclic AIPs

Species	Genetic locus	AIP	
***S. aureus***	*agrBDCA*	AIP-1	YST[CDFIM]*
		AIP-2	GVNA[CSSLF]
		AIP-3	IN[CDFLL]
		AIP-4	YST[CYFIM]
***S. epidermidis***	*agrBDCA*	AIP-1	DSV-[CASYF]
		AIP-2	NASKYNP-[CSNYL]
		AIP-3	NAAKYNP-[CASYL]
***Staphylococcus lugdunensis***	*agrBDCA*	AIP-1	DI-[CNGYF]
***Staphylococcus hyicus***	*agrBDCA*	AIP	KINP-[CTVFF]
***Staphylococcus haemolyticus***	*agrBDCA*	AIP	SFTP-[CTTYF]
***S. pseudintermedius***	*agrBDCA*	AIP	RIPT[STGFF]
***Clostridium acetobutylicum***	*agrBD agrCA*	AIP	[CVLVTL]
***Clostridium botulinum***	*agrB1D1*	AIP-1	ACYW[CVYQP]
	*agrB2D2*	AIP-2	ADSA[CHLGI]
***Clostridioides difficile***	*agrD1B1*		ANST[CPWII]
	*agrB2D2C2A2*		NSA[SSWVA]
***C. perfringens***	*agrBD*	AIP	[CLWFT]
***Lactobacillus plantarum***	*lamBDCA*	AIP	[CVGIW]
***L. monocytogenes***	*agrBDCA*	AIP	A[CFMFV]
***E. faecalis***	*fsrBDCA*	GBAP	QN[SPNIFGQWM]

* Square brackets denote amino acid residues within the AIP macrocycle.

## The *agr* system

**Fig. 1.**
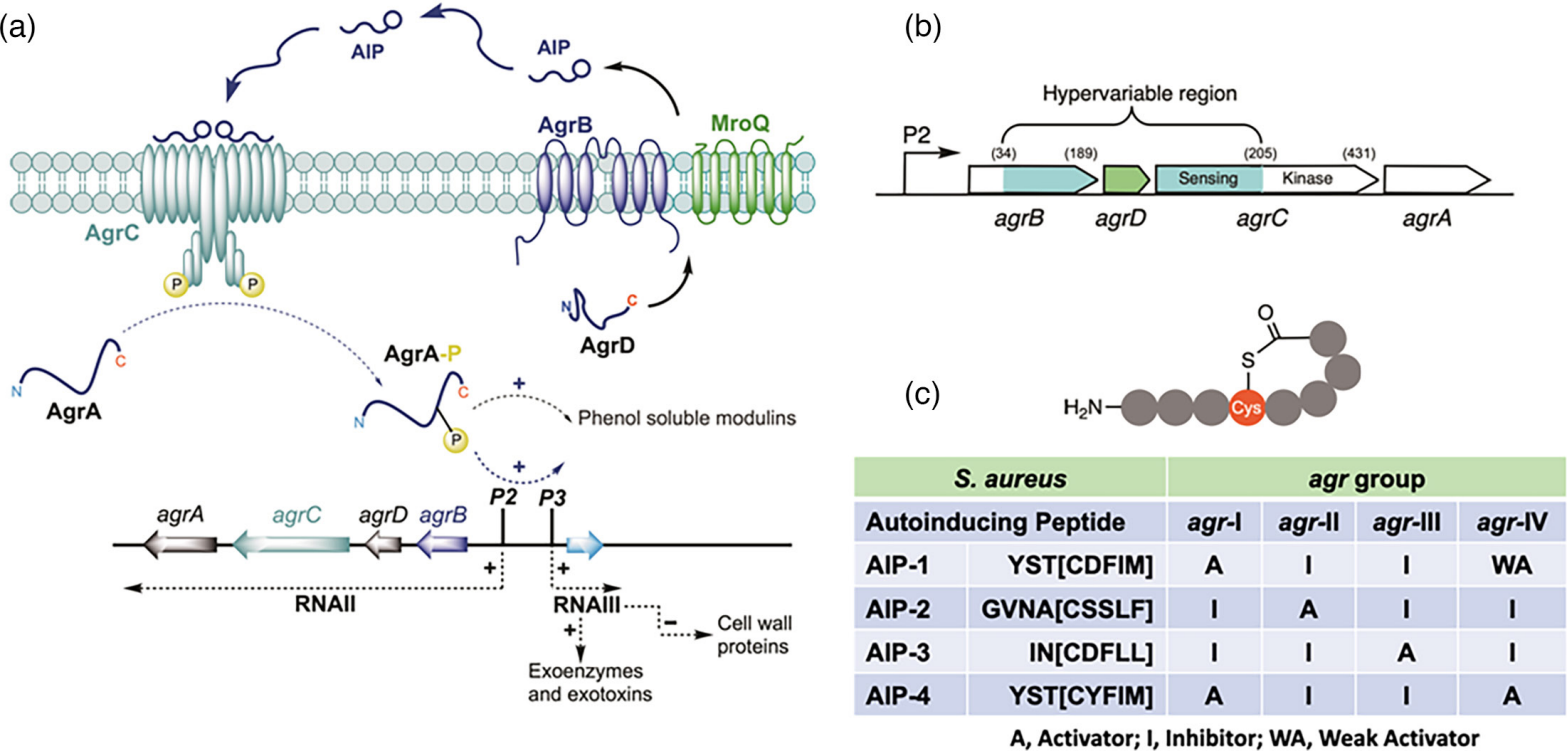
(**a**) Schematic of the staphylococcal *agr* QS system. The *agrBDCA* locus is composed of two divergent transcripts, RNAII and RNAIII, driven by the *agr*P2 and *agr*P3 promoters, respectively. AgrD, the pro-peptide precursor of the AIP, is processed at the cytoplasmic membrane by AgrB and MroQ such that AIPs are released extracellularly. AIPs bind to and activate the AgrC receptor, a membrane-bound histidine sensor kinase, resulting in phosphorylation of the response regulator AgrA and activation of the *agr*P2 and *agr*P3 promoters. This drives the autoinduction circuitry to generate more AIP signal molecules and induces expression of virulence genes either indirectly via RNAIII or directly via the target gene promoters. (**b**) Schematic showing the hypervariable region (in green/cyan) of the *agrBDCA* locus incorporating *agrD* and giving rise to the different *agr* groups. Amino acid residues marking the beginning and ends of the variable regions are numbered. (**c**) Generalized AIP structure and summary table showing the amino acid sequences of the AIPs belonging to each of the 4 S. *aureus agr* groups and their cross-group activities. The square brackets denote the amino acid residues within the macrocycle. In *S. aureus*, the AIP N-terminal tails have 2, 3 or 4 aa residues (reproduced from further reading reference 2).

The versatility of *Staphylococcus aureus* as a pathogen revolves around diverse cell wall colonization factors, immune modulating agents and exotoxins, many of which are controlled via the *agr* system. While the cell wall proteins are characteristically expressed in the early exponential phase and later down-regulated, multiple exotoxins are up-regulated in the post-exponential phase. The prototypical *S. aureus agr* locus reciprocally controls and co-ordinates the expression of these virulence factors. In *S. aureus* and the coagulase-negative staphylococci (CoNS, such as *Staphylococcus epidermidis*), the *agr* locus consists of two divergent transcriptional units, *agrBDCA* and a regulatory RNA effector (RNAIII), controlled, respectively, by the *agr*P2 and *agr*P3 promoters (Fig. 1). AgrA and AgrC form a two-component system (TCS) that incorporates a histidine sensor kinase (AgrC) and a cytoplasmic response regulator DNA-binding protein (AgrA). The *agrD* gene codes for a pro-peptide from which the QS signal (an AIP) is enzymatically generated via AgrB (Fig. 1). RNAIII is a large (514 nt) multifunctional regulatory RNA that functions mainly by inhibiting translation initiation of target genes and influencing mRNA translation and stability either directly or through inhibition of both positive and negative transcriptional regulators. It also codes for a small haemolysin, *δ*-toxin. The predicted transmembrane nature of the sensor AgrC suggested that there might be a small molecule extracellular activator of AgrC likely present in spent culture supernatants of the parent but not the *agr* mutant. This was confirmed, and the putative QS signal molecule was discovered to be a cyclic AIP (Fig. 1) derived from the AgrB-dependent processing of the ribosomally synthesized AgrD pro-peptide. Structurally, staphylococcal AIPs are thiolactones of 7–9 aa residues in which the thiol (SH group) of the central cysteine is linked to the C-terminal amino acid of the peptide, forming a ring incorporating 5 aa residues with an exocyclic N-terminal extension of variable length (Fig. 1).

The *S. aureus agr* locus is polymorphic with four allelic variants (termed groups I–IV) that incorporate a hypervariable region within the *agrB*, *agrD* and *agrC* genes (Fig. 1). This gives rise to four AIPs, which differ in their primary amino acid sequences and explain their specificity for AgrC as activators (agonists) of their cognate AgrC receptors but competitive inhibitors (antagonists) of the other AgrC variants. For example, AIP-1 is an agonist of AgrC1 but an antagonist of the AIP-2/AgrC2 and AIP-3/AgrC3 interactions, whereas AIP-2 and AIP-3 both inhibit AIP-1/AgrC1 interactions (Fig. 1). This cross-inhibition has given rise to the concept of ‘competitive interference’. Analysis of some 40,000 *S*. *aureus* genomes has revealed that the distribution of the four *agr* groups is ~60% group I, ~22% group II, ~14% group III and ~2.5% group IV. There do not, however, appear to be any clear relationships between *agr* groups and specific types of *S. aureus* infection.

**Fig. 2.**
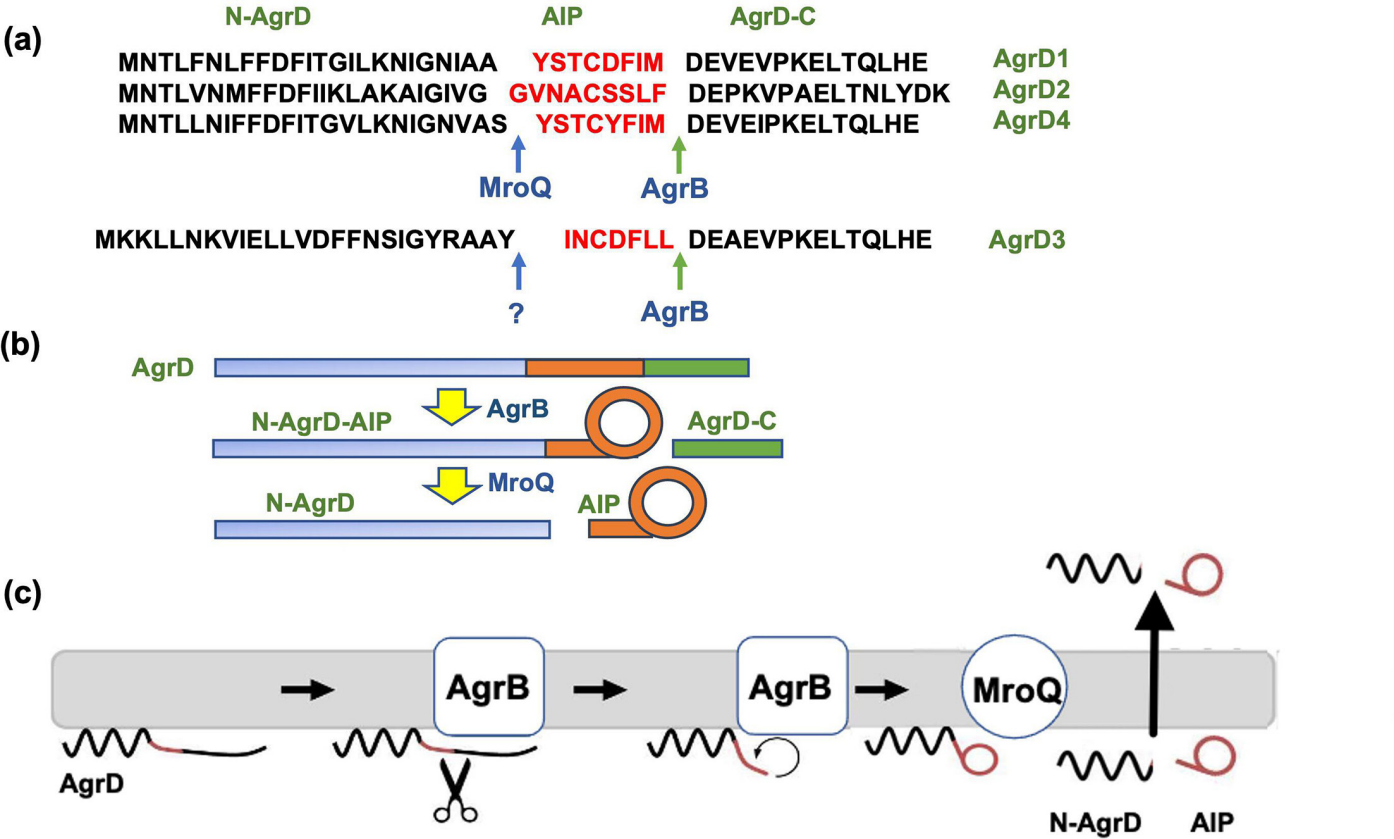
Schematic showing the processing of AgrD pro-peptides to generate the active cyclic AIP signal molecules. (**a**) Amino acid sequences of AgrD1–D4 showing the AgrB cleavage site and the MroQ sites for AgrD1, D2 and D4. MroQ does not cleave AgrD3. (**b**) Processing of AgrD by AgrB and MroQ to release N-AgrD and the cyclic AIP. (**c**) Schematic showing the formation and release of the AIP and N-AgrD at the cytoplasmic membrane. Cleavage of AgrD by AgrB releases a 14 aa C-terminal peptide (AgrD-C), which is degraded in the cytoplasm. N-AgrD-AIP is cleaved by MroQ to release N-AgrD and the mature AIP. The mechanism by which the AIP and N-AgrD are exported is not known (reproduced from further reading reference 2).

The AgrD pro-peptide consists of 40–50 aa residues, which can be divided into three regions: an N-terminal amphipathic leader (24–25 residues), a mid ‘AIP’ region of 7–9 aa residues and a charged C-terminal tail (14–15 residues). In *S. aureus*, AIP generation requires at least two transmembrane proteases (AgrB and MroQ) and four steps: removal of the C-terminal tail of AgrD, formation of the thiolactone by AgrB, cleavage of the N-terminal leader peptide by MroQ and export of the AIP (Fig. 2). Given the hypervariable region present in AgrB, it is perhaps not surprising that AgrB, in common with AgrC, exhibits some substrate specificity in that AgrB1 can process AgrD1 and AgrD2 but not AgrD3 and vice versa. The gene coding for *mroQ* is located upstream of the *S. aureus agr* locus. MroQ is a member of the CAAX protease and bacteriocin-processing enzyme family of membrane metalloproteases. Although the MroQ amino acid sequence does not vary with *agr* group, in enzyme assays, MroQ processed the *agr* groups I and II but not the group III peptide thiolactone intermediates to form the respective AIP, implying the existence of alternative enzymes. Both the AIP and the N-terminal AgrD leader peptide are released extracellularly, although the export mechanism involved has yet to be elucidated.

The histidine kinase sensor AgrC is a transmembrane protein consisting of an N-terminal sensory domain containing a putative externally located AIP-binding site linked to a C-terminal cytoplasmic region incorporating a dimerization and histidine phosphorylation sub-domain and a catalytic and ATP-binding sub-domain. AgrC/AIP interactions are highly specific, occurring at nanomolar affinities. Upon binding to AgrC, the cognate AIP induces *trans*-auto-phosphorylation, resulting in the transfer of a phosphate to AgrA. This then binds to the *agr*P2 promoter, upregulating *agrBCDA* and establishing a positive-feedback loop that autoinduces AIP synthesis and drives downstream gene expression via the AgrA-dependent activation of *agr*P3 and the regulatory RNA, RNAIII or directly via AgrA-binding to target promoters (Fig. 1). AIPs are active at nanomolar concentrations, and their exogenous provision at the time of inoculation can overcome the quorum, i.e. prematurely induce the *agr* system, although there is a ‘window’ after which *S. aureus* no longer responds. As with AHL-dependent QS, the *agr* ‘master’ virulence regulator is subject to further controls via an interconnected network of regulatory elements (including TCSs, sigma factors and repressors). These facilitate the integration of diverse environmental and host cues and may well contribute to the ‘window’ period of AIP responsiveness.

Extensive AIP (structure activity relationships, SAR) relationship studies using transcriptional reporters in *S. aureus* in combination with native AIPs and AIP analogues have established the key structural features required for AgrC activation and inhibition. Changes in the AIP amino acid sequence or isomer, altering the ring size or removing the exocyclic amino acid residues, are not tolerated, and the corresponding linear peptides are inactive. AIP binding and receptor activation require both the macrocycle and exocyclic regions. In contrast, competitive inhibition is much more tolerant to AIP sequence variation but still requires the AIP macrocycle peptide.

## *agr* and host interactions

In experimental animal models of respiratory, arthritis, skin, bone and soft tissue infections, *S. aureus agr* mutants are attenuated, whereas, in contrast, the *agr* system is dispensable in chronic, biofilm-associated infections. The ability to switch *agr* on or off during different stages of infection of host cells and in different tissues is likely advantageous when moving from an acute toxigenic to a chronic persistent lifestyle. By forming a biofilm or taking up intracellular residence, *S. aureus* can avoid host cellular immune defences. The subsequent escape from an intra-cellular endosomal niche has been termed ‘confinement’ or ‘compartment’ sensing rather than QS since *agr* is being activated by a single, trapped cell in response to its own AIP rather than responding to AIPs made by other cells in a population.

Despite the relevance of *agr* to *S. aureus* virulence in experimental animal infection models, *agr*-defective mutants are commonly found in clinical samples from both asymptomatic nasal carriage and serious infections. *agr* dysfunction has been strongly associated with hospitalization and antibiotic usage, suggesting a trade-off between virulence and antibiotic resistance. Interestingly, a study of over 40,000 *S. aureus* genomes failed to discover any stable *agr*-defective strain lineages. It is therefore possible that *agr*-defective strains may be unable to establish and maintain circulating populations outside their original infection sites. Such *agr* mutants are, however, capable of prolonged intracellular survival, even though they cannot escape from endosomes or phagosomes.

These findings also imply that *S. aureus* populations progressing from colonization to infection at different body sites may be heterogeneous with respect to *agr* expression or consist of a mixed population of *agr*-functional and *agr*-defective mutants or even contain a fraction of phase variable cells capable of reverting to *agr*-functional status. DNA sequence analysis of *S. aureus agr*-dysfunctional clinical isolates has revealed frameshifts, insertions, deletions and substitutions in the *agrBDCA* operon – mostly in *agrC* and some in *agrA*. This frequent occurrence of common but independently acquired mutations may be an adaptive response to specific host selective pressures. Some of these natural mutations result in the complete loss of *agr* functionality, while others impact the timing or strength of *agr* induction. Whether increasing the threshold for *agr* activation offers a fitness advantage remains to be established.

Host factors may also impact the *agr*-driven switch from a colonizing to an invasive phenotype. For example, serum proteins such as the low-density and very-low-density particle-associated apo-lipoprotein B (ApoB) interfere with *agr*-dependent QS by specifically and reversibly sequestering AIPs. The relevance of these findings *in vivo* is related to the increased susceptibility of mice deficient in ApoB to invasive infection by an *S. aureus* wild-type strain compared with an isogenic *agr* mutant. AIP-1 and AIP-4 both incorporate a methionine residue within their rings, which undergoes S-oxidation in response to phagocyte-derived reactive oxygen and nitrogen species. This results in AIP inactivation, resulting in the down-regulation of *agr* and the reduction of virulence in a mouse skin infection model. Oxidative stress also down-regulates *agr* through AgrA inactivation, which drives the emergence of *agr* mutants. Consequently, *agr* imposes an oxygen-dependent fitness cost such that hypoxia favours maintenance of QS and increased exotoxin production. Consequently, this oxygen-driven tuning of the *agr* system may exert a major influence on tissue-dependent disease progression during infection.

## Variations on a theme: *agr* beyond *S. aureus*

Apart from *S. aureus*, the *agr* system is present in the CoNS, clostridia, enterococci, *Listeria* and *Lactobacillus* (Table 1). The CoNS are a diverse group of staphylococcal species primarily found on the healthy skin and mucous membranes of humans and other mammals. The *agrBDCA* genes and RNAIII are widespread in the CoNS but highly divergent. Based on their AgrD peptide sequences, most CoNS, in common with *S. aureus*, can be divided into different *agr* sub-groups – between 2 and 6, depending on the species. *S. epidermidis,* for example, has four *agr* groups. CoNS species produce AIPs that, in common with *S. aureus*, are usually between 7 and 9 aa residues and incorporate a thiolactone ring. The exceptions are *S. pseudintermedius*, which produces an AIP lactone (rather than a thiolactone, since the characteristic AIP central cysteine has been replaced by a serine residue, resulting in replacement of the ring S by O), and *S. epidermidis*, which makes AIPs with extended exocyclic tails. Competitive interference also occurs within and between different CoNS species and with *S. aureus*.

Multiple *agr* variants and AIPs have also been described in *Clostridium* species. These are involved in regulating sporulation, virulence, butanol production, motility and biofilm formation. Similarly, an *agr* system regulating virulence, chitinase and biofilm development has been described for *Listeria monocytogenes*. In both genera, an associated regulatory RNA functionally equivalent to RNAIII is absent. The organization of the clostridial *agr* systems also differs from the staphylococci, with some lacking *agrC* and *agrA* genes and others where *agrBD* and *agrCA* form separate operons. In addition, some AIPs incorporate macrocycle peptides with 6 rather than 5 aa residues, while others lack N-terminal exocyclic extensions, and these can undergo re-arrangement reactions to form different ring structures. The largest AIP macrocycle described to date is the 11 aa residue AIP incorporating a 9 residue macrocycle produced by *Enterococcus faecalis*. Termed GBAP (gelatinase biosynthesis activating peptide), this AIP is a lactone rather than a thiolactone since the characteristic central cysteine of the staphylococcal AIPs has been replaced by a serine residue. The *E. faecalis* locus responsible for GBAP production (*fsrBDCA*) is similar to the staphylococcal *agrBDCA* locus, but it lacks RNAIII such that transcriptional activation occurs exclusively via FsrA. Fsr plays important roles in biofilm development, persistence and immune evasion and is required for virulence in several infection models.

## Exploiting *agr*

The *agr*-dependent regulation of virulence and cross-group interference in *S. aureus* has highlighted the therapeutic potential of small molecule AgrC inhibitors as anti-virulence agents. In a mouse skin abscess infection model, treatment with synthetic AIP-2 attenuated infection caused by an *agr* group I *S. aureus* strain. Extensive SAR studies have resulted in the synthesis of highly potent *agr* inhibitors for *S. aureus*, *L. monocytogenes*, *C. perfringens* and *E. faecalis*. In addition to AgrC, AgrA and AgrB are also druggable targets for which inhibitory compounds have been identified. However, much more *in vivo* work will be required before any *agr* inhibitors with the appropriate pharmacological properties can enter human clinical trials. These are likely to be complicated given the *agr* dysfunction associated with *S. aureus* clinical isolates and the *agr* switching that occurs during different stages of infection. However, many of the AIPs of many different CoNS species competitively inhibit the *S. aureus agr* system and have potential for treating skin conditions such as atopic dermatitis (AD). *S. aureus* colonizes AD patients’ lesions, driving inflammation and degradation of the skin’s barrier function. Employing antagonistic non-pathogenic CoNS strains or their AIPs could offer a realistic means of preventing *S. aureus-*mediated AD exacerbations.

## Further reading

1. **Neiditch MB, Capodagli GC, Prehna G, Federle MJ**. Genetic and structural analyses of RRNPP intercellular peptide signaling of Gram-positive bacteria. *Annu Rev Genet*. 2017;51 : 311–333.
2. **Williams P, Hill P, Bonev B, Chan WC**. Quorum-sensing, intra- and inter-species competition in the staphylococci. *Microbiology (Reading*). 2023;169 : 001381.
3. **Bodine SP, Muir TW**. Molecular mechanisms of virulence regulation in *Staphylococcus aureus*: A journey into reconstitutive biochemistry. *Acc Chem Res*. 2025;58 : 1657–1669.
4. **Ahmed UKB, Ballard JD**. Autoinducing peptide-based quorum signaling systems in *Clostridioides difficile. Curr Opin Microbiol*. 2022;65 : 81–86.
5. **West KHJ, Ma SV, Pensinger DA**, *et al*. Characterization of an autoinducing peptide signal reveals highly efficacious synthetic inhibitors and activators of quorum sensing and biofilm formation in *Listeria monocytogenes. Biochemistry*. 2023;62 : 2878–2892.
6. **Gless BH, Bejder BS, Monda F, Bojer MS, Ingmer H, Olsen CA**. Rearrangement of thiodepsipeptides by S → N acyl shift delivers homodetic autoinducing peptides. *J Am Chem Soc*. 2021;143 : 10514–10518.
7. **McBrayer DN, Cameron CD, Gantman BK, Tal-Gan Y**. Rational design of potent activators and inhibitors of the *Enterococcus faecalis fsr* quorum sensing circuit. *ACS Chem Biol*. 2018;13 : 2673–2681.
8. **Parlet CP, Brown MM, Horswill AR**. Commensal staphylococci influence *Staphylococcus aureus* skin colonization and disease. *Trends Microbiol*. 2019;27 : 497–507.
9. **Gallo RL, Horswill AR**. *Staphylococcus aureus*: The bug behind the itch in atopic dermatitis. *J Invest Dermatol*. 2024;144 : 950–953.
10. **Otto M**. Critical assessment of the prospects of quorum-quenching therapy for *Staphylococcus aureus* infection. *Int J Mol Sci*. 2023;24 : 4025.

## Abbreviations

AD: atopic dermatitis
*agr*: accessory gene regulation
AHL: *N*-acylhomoserine lactone
AIP: autoinducing peptide
CoNS: coagulase-negative staphylococci
GBAP: gelatinase biosynthesis activating peptide
QS: quorum sensing
SAR: structure activity relationships
TCS: two-component system

